# Supplementary figures and images for: FGF19 and its analog Aldafermin cooperate with MYC to induce aggressive hepatocarcinogenesis
Source: EMBO Mol Med. 2024 Jan 16;16(2):2. doi: 10.1038/s44321-023-00021-x (PMC10897482; doi:10.1038/s44321-023-00021-x)

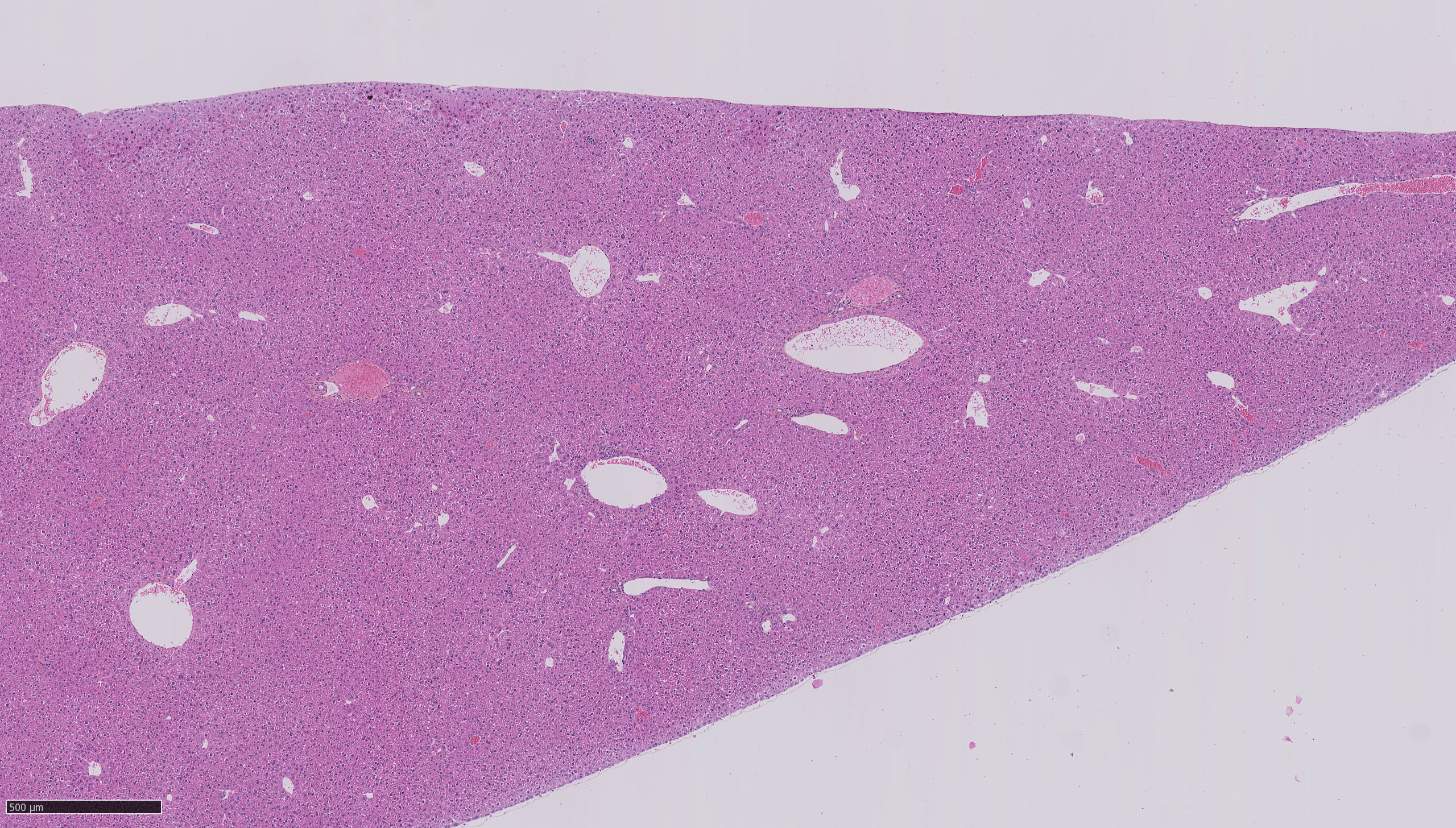

Supplement: Supplementary file 2 — Source Data Fig. 1 [file 44321_2023_21_MOESM2_ESM.zip › Figure 01 SourceData/Fig01PanelB/Mouse#1 - HES.jpg]

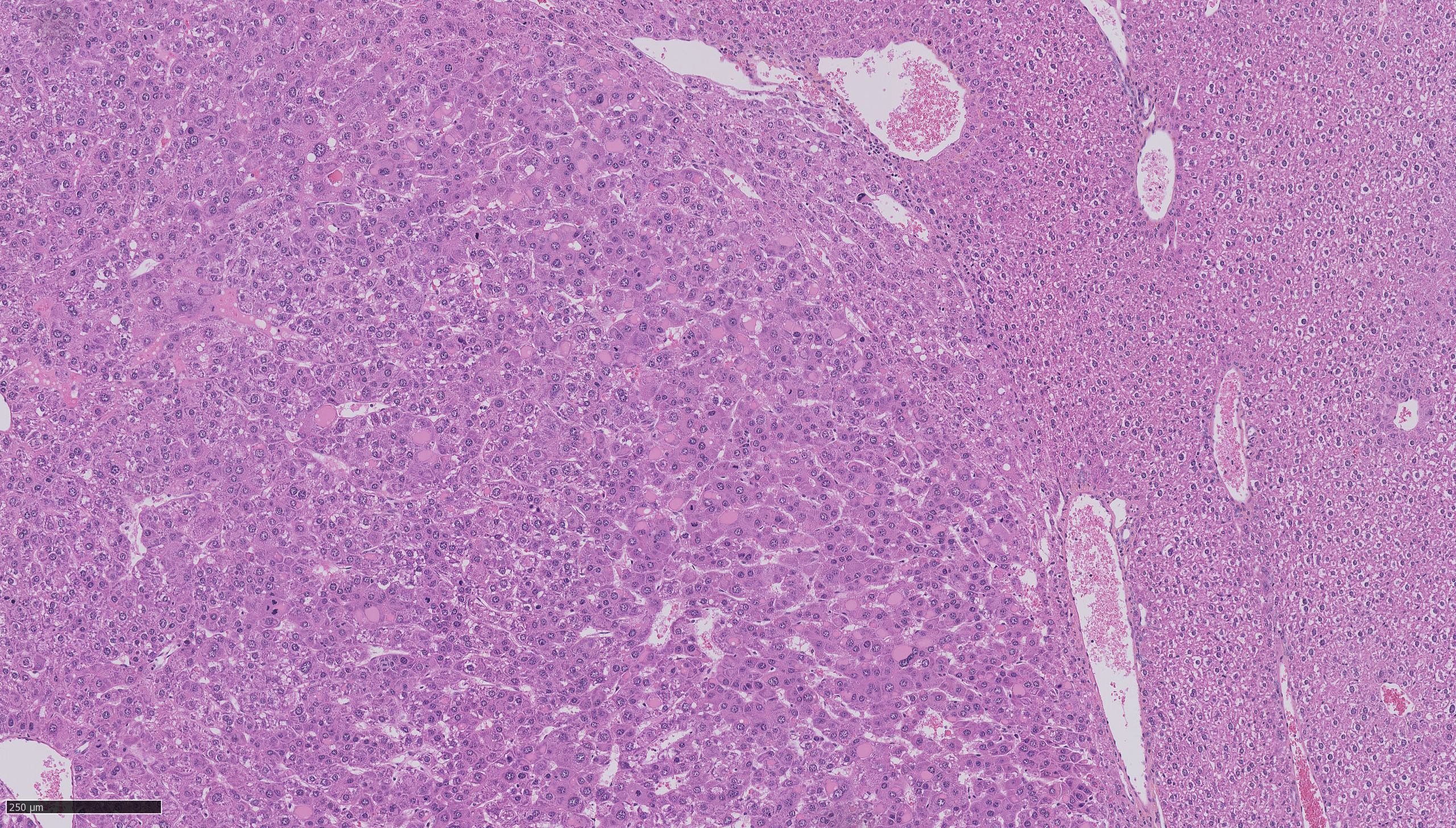

Supplement: Supplementary file 2 — Source Data Fig. 1 [file 44321_2023_21_MOESM2_ESM.zip › Figure 01 SourceData/Fig01PanelB/Mouse#19 - HES.jpg]

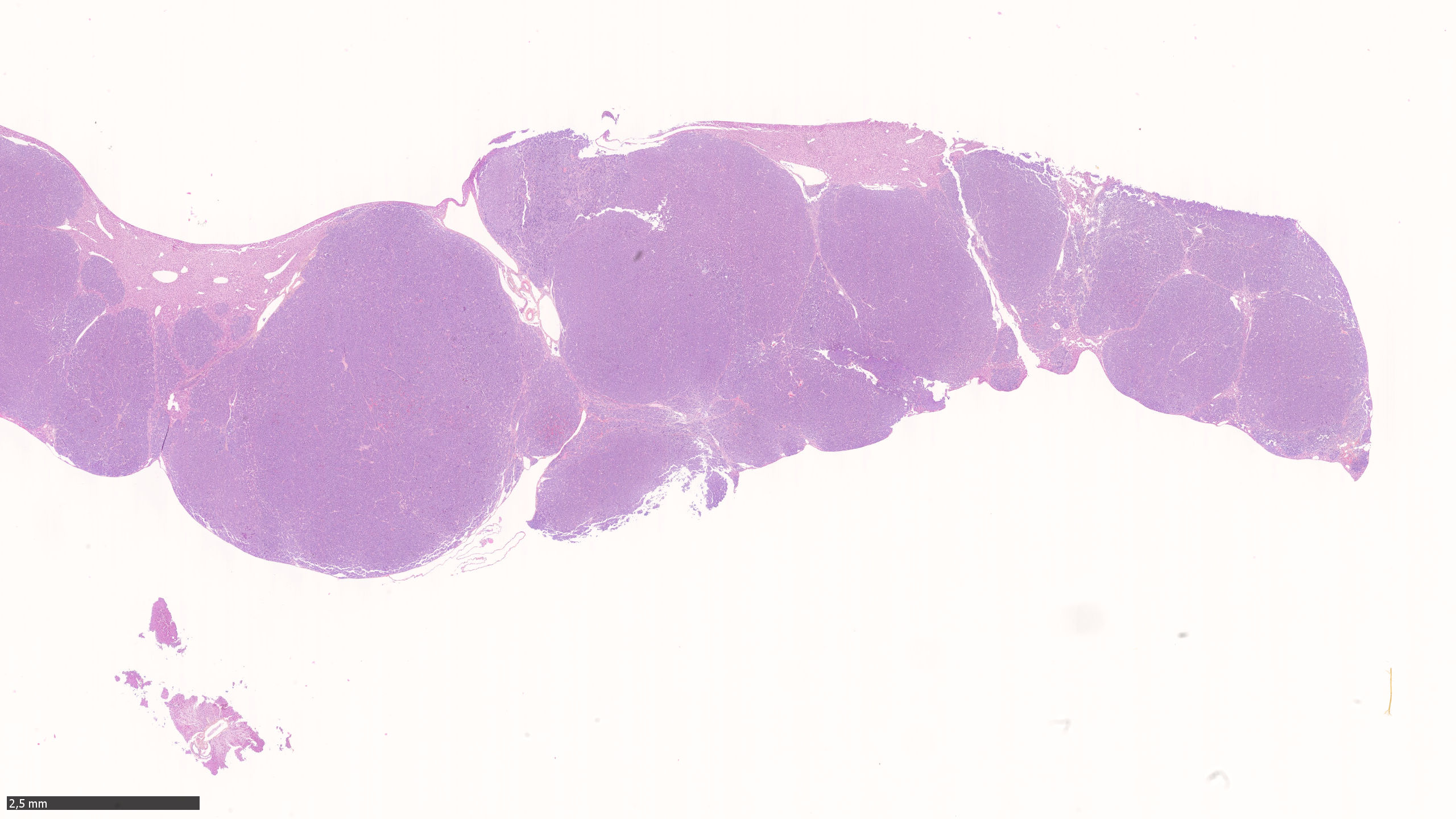

Supplement: Supplementary file 2 — Source Data Fig. 1 [file 44321_2023_21_MOESM2_ESM.zip › Figure 01 SourceData/Fig01PanelD/Mouse#100_export_1.25_2x.jpg]

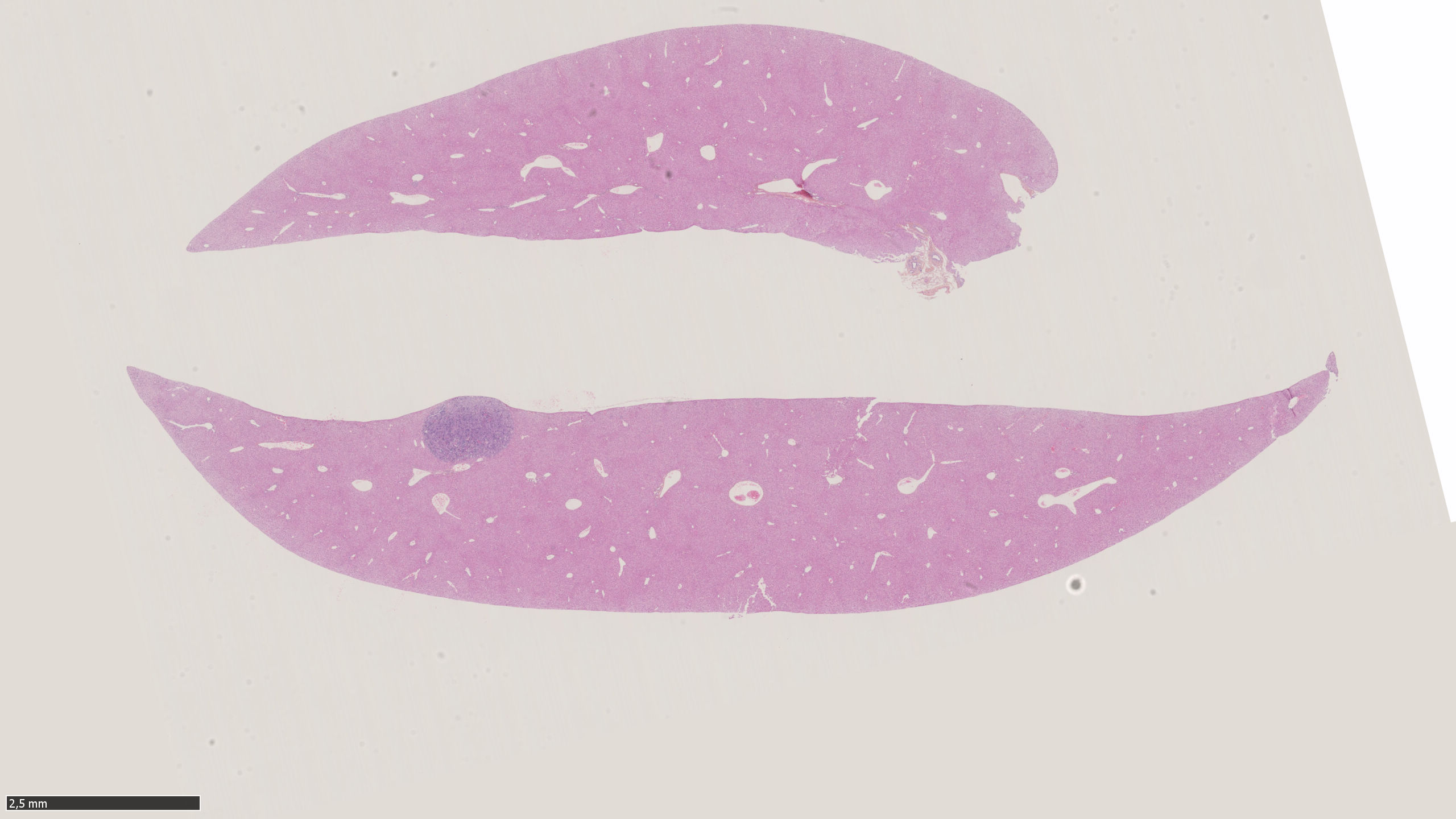

Supplement: Supplementary file 2 — Source Data Fig. 1 [file 44321_2023_21_MOESM2_ESM.zip › Figure 01 SourceData/Fig01PanelD/Mouse#57_export_1.25x.jpg]

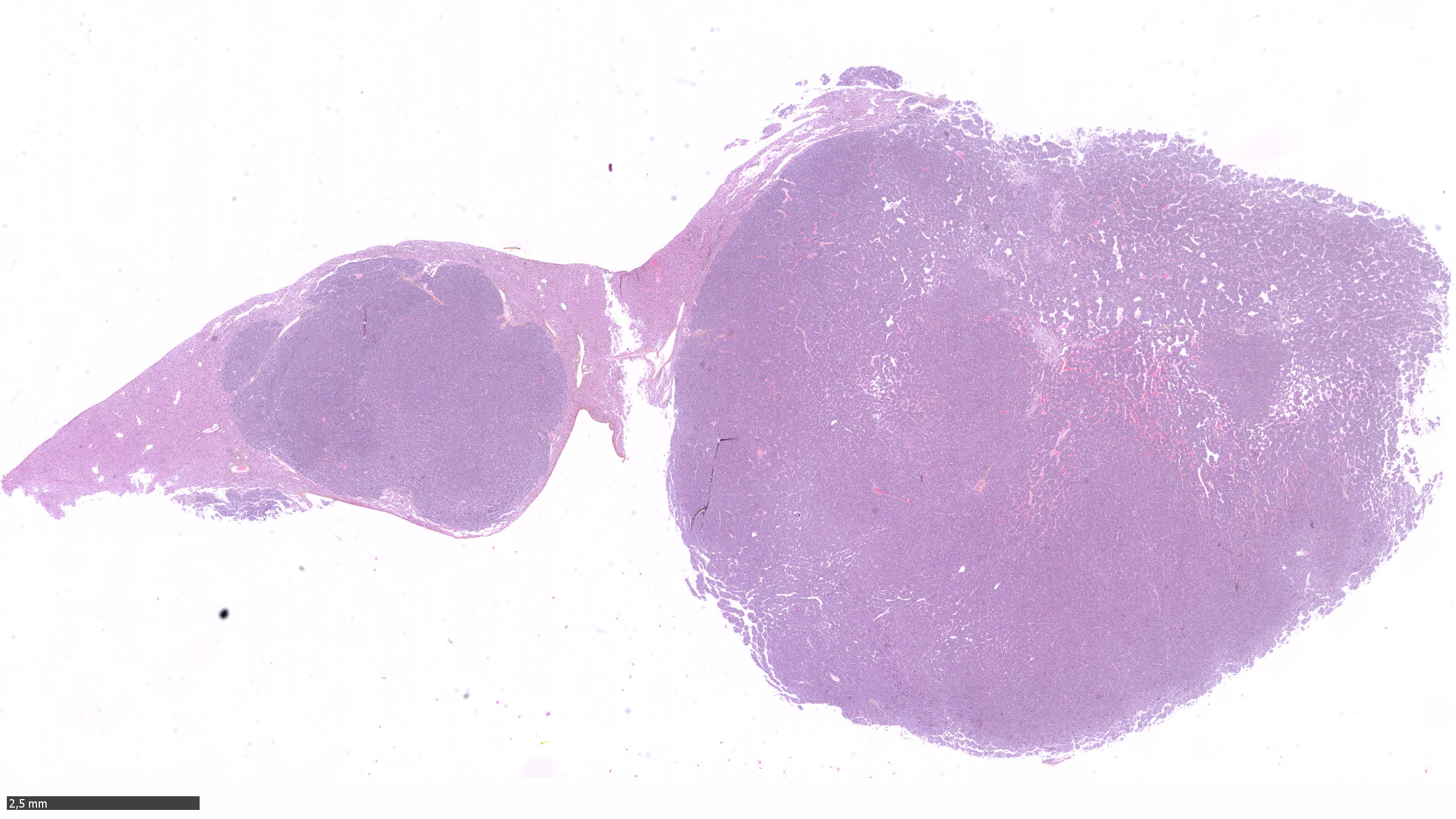

Supplement: Supplementary file 2 — Source Data Fig. 1 [file 44321_2023_21_MOESM2_ESM.zip › Figure 01 SourceData/Fig01PanelD/Mouse#71_export_1.25x.jpg]

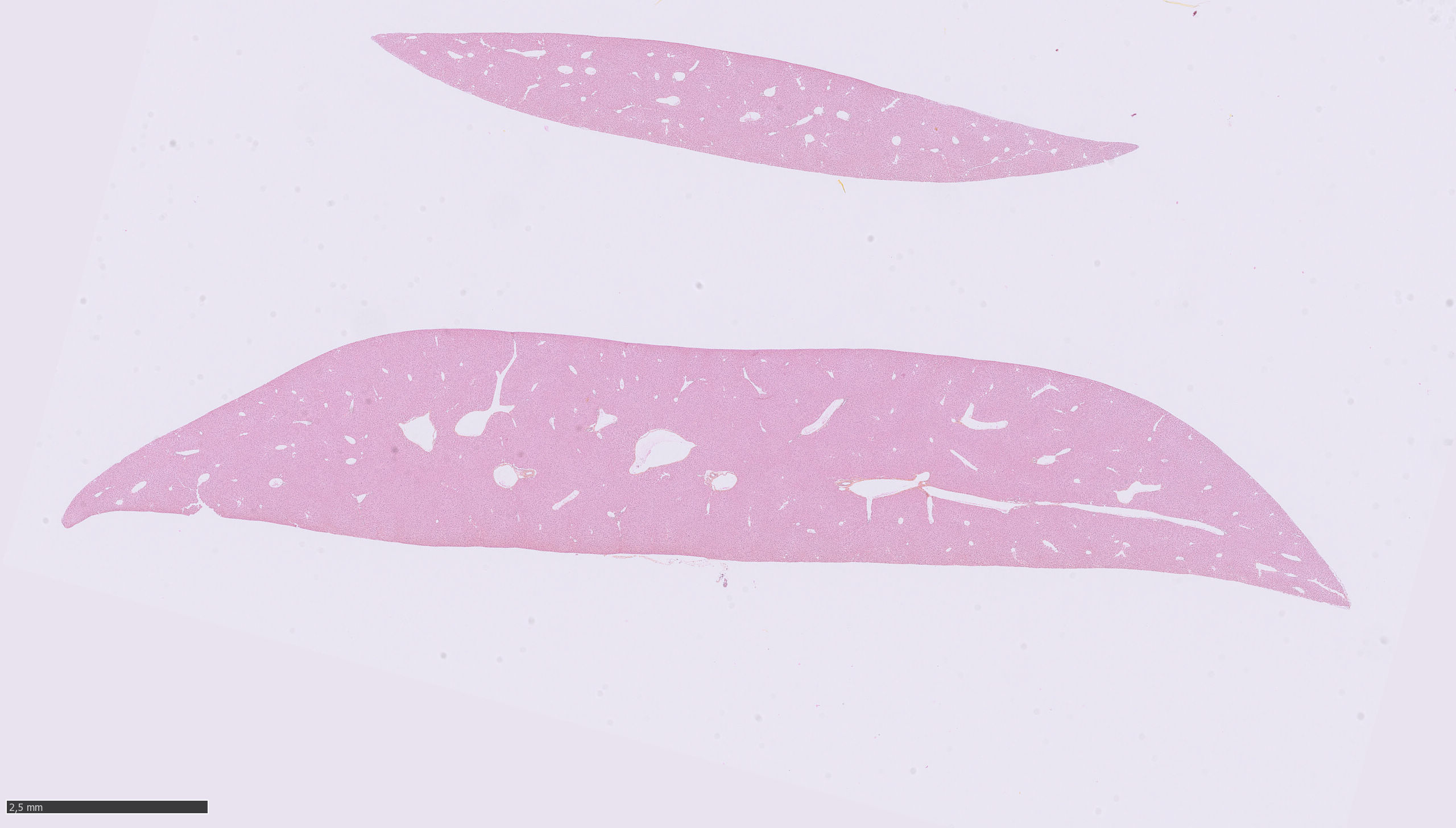

Supplement: Supplementary file 3 — Source Data Fig. 2 [file 44321_2023_21_MOESM3_ESM.zip › Figure 02 SourceData/Fig02PanelB/Mouse#109 - HES.jpg]

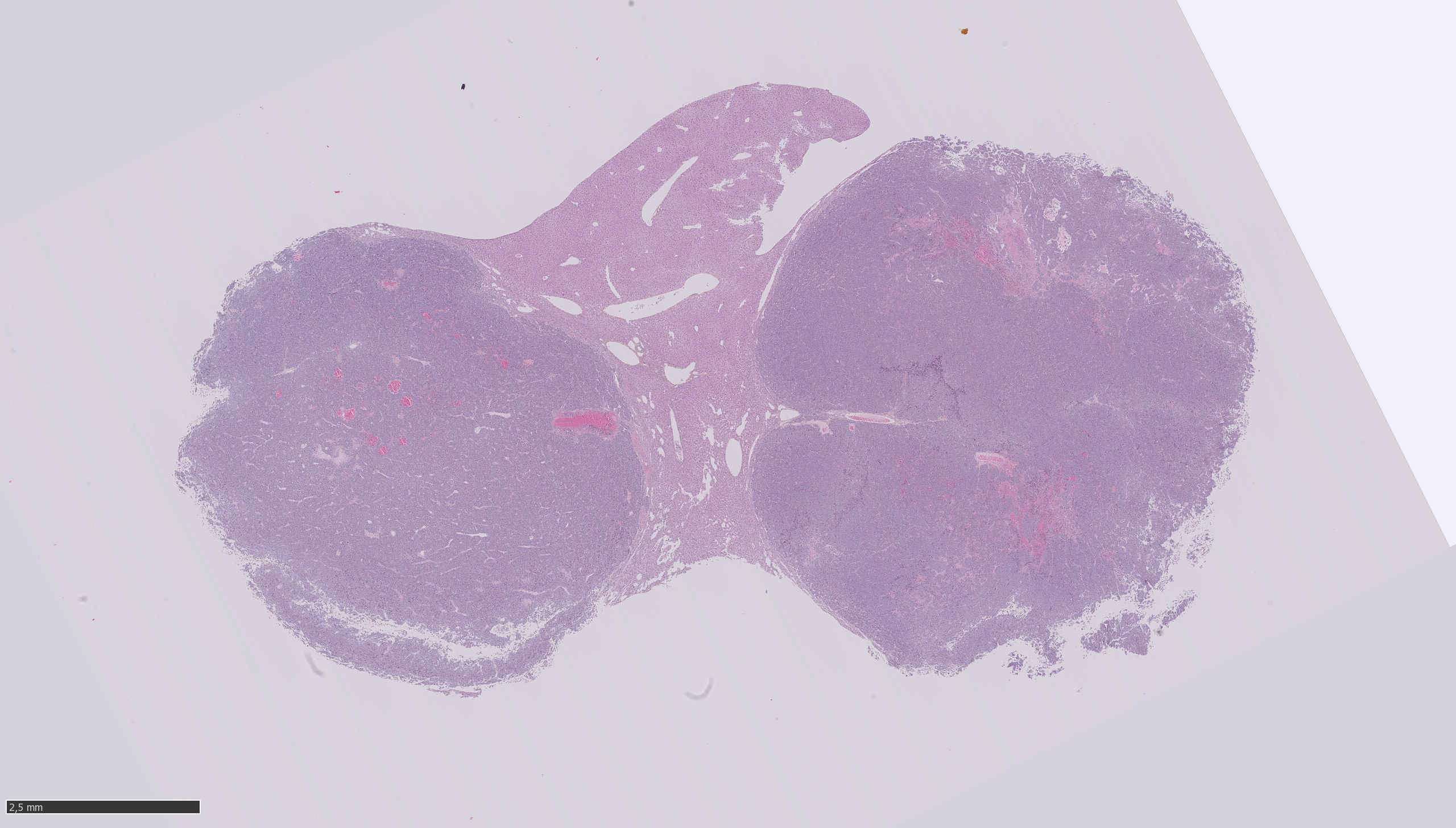

Supplement: Supplementary file 3 — Source Data Fig. 2 [file 44321_2023_21_MOESM3_ESM.zip › Figure 02 SourceData/Fig02PanelB/Mouse#124 - HES.jpg]

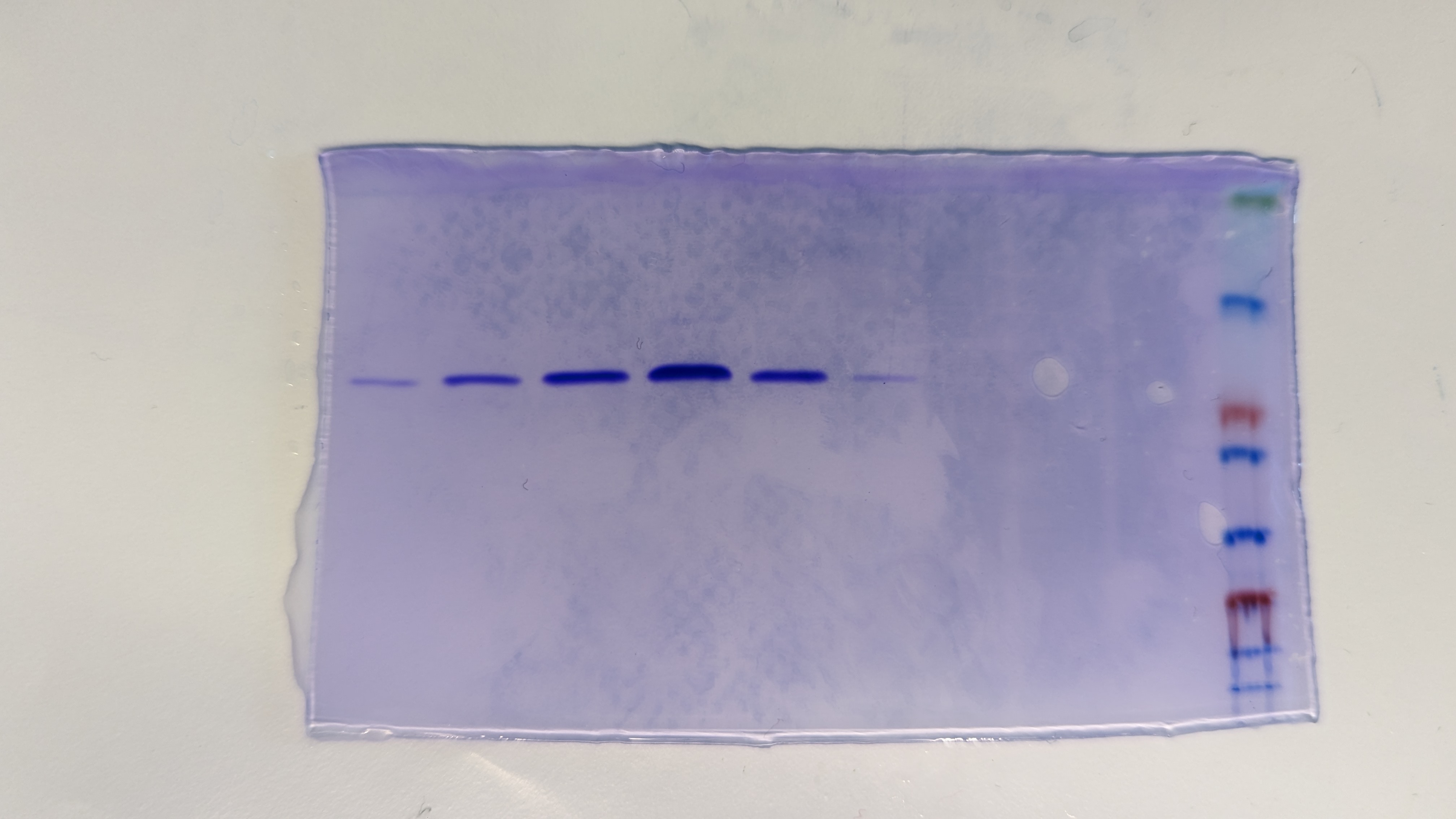

Supplement: Supplementary file 4 — Source Data Fig. 3 [file 44321_2023_21_MOESM4_ESM.zip › Figure 03 SourceData/Fig03PanelA/Fig03PanelA - Purification FGF19.jpg]

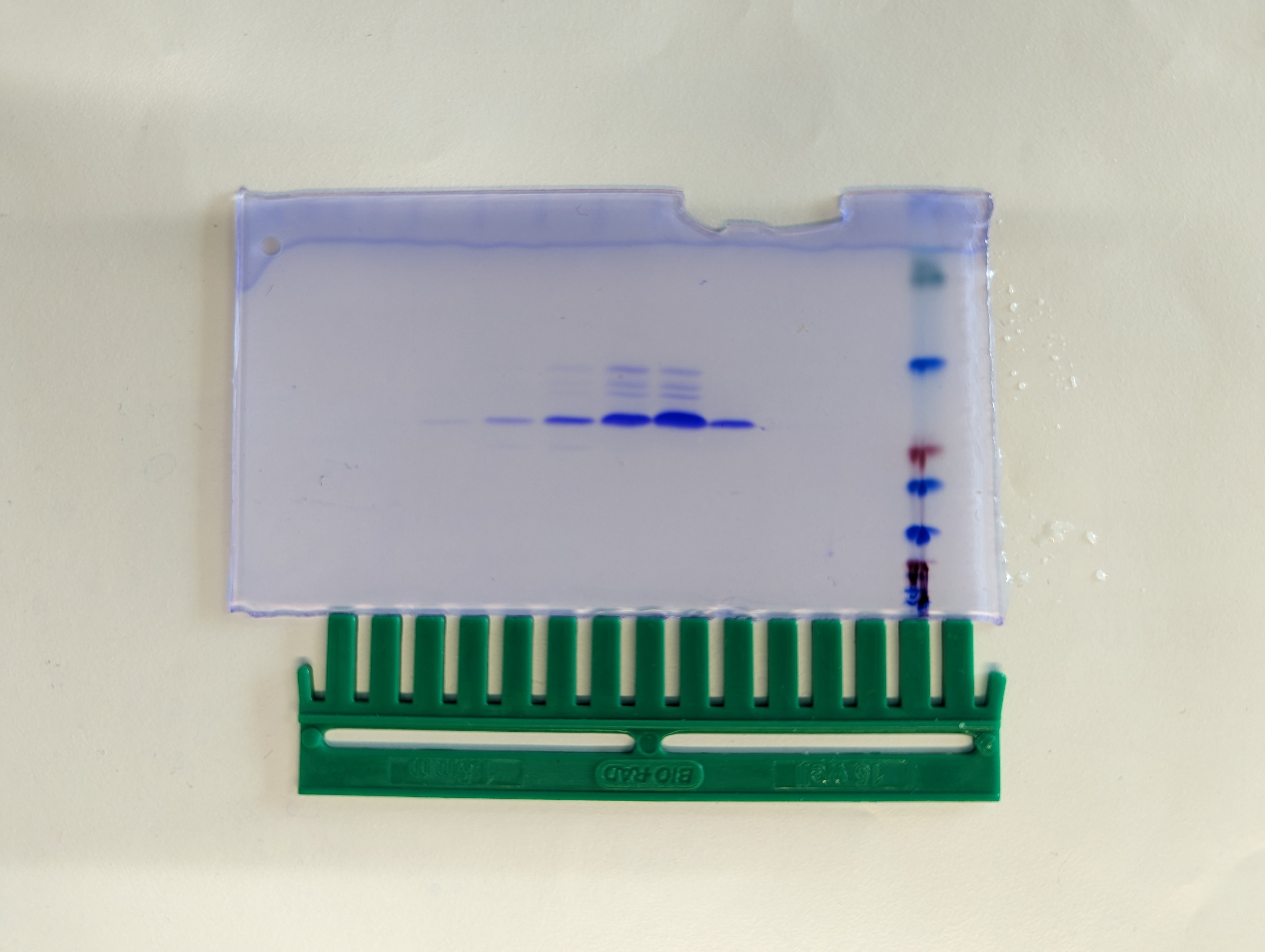

Supplement: Supplementary file 4 — Source Data Fig. 3 [file 44321_2023_21_MOESM4_ESM.zip › Figure 03 SourceData/Fig03PanelA/Fig03PanelA - PurificationAldafermin.jpg]

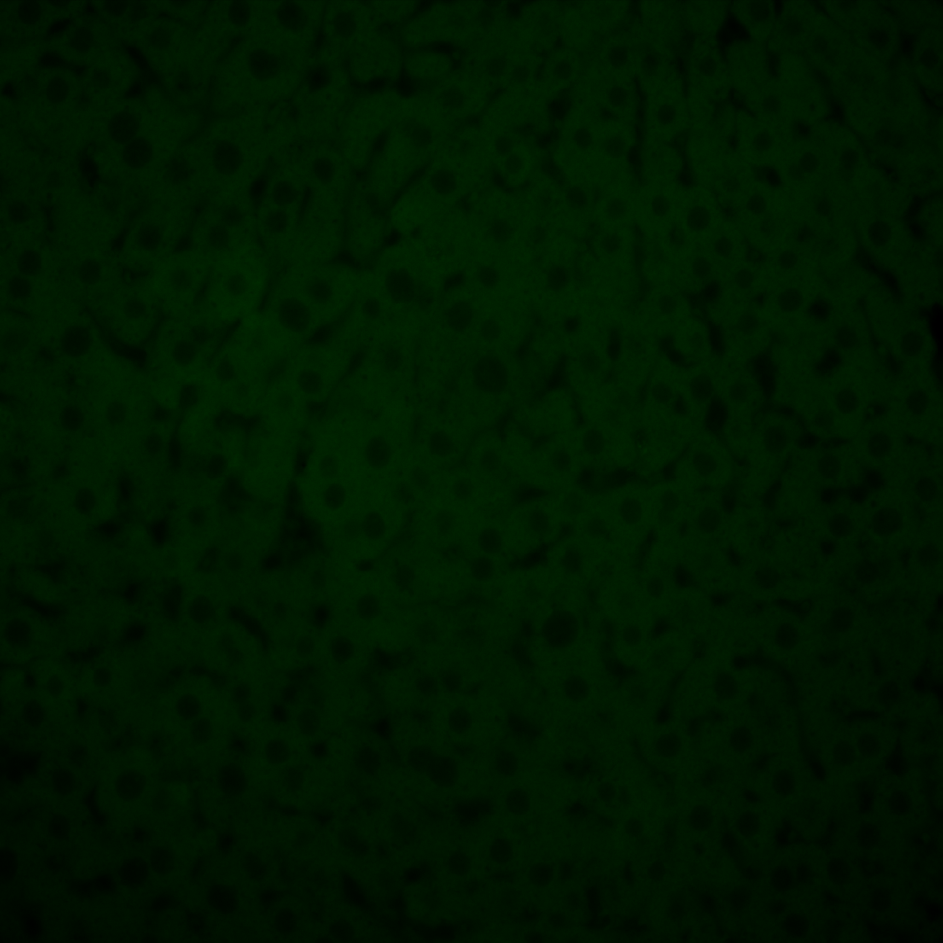

Supplement: Supplementary file 4 — Source Data Fig. 3 [file 44321_2023_21_MOESM4_ESM.zip › Figure 03 SourceData/Fig03PanelC/Mouse#151-40x-G.tif]

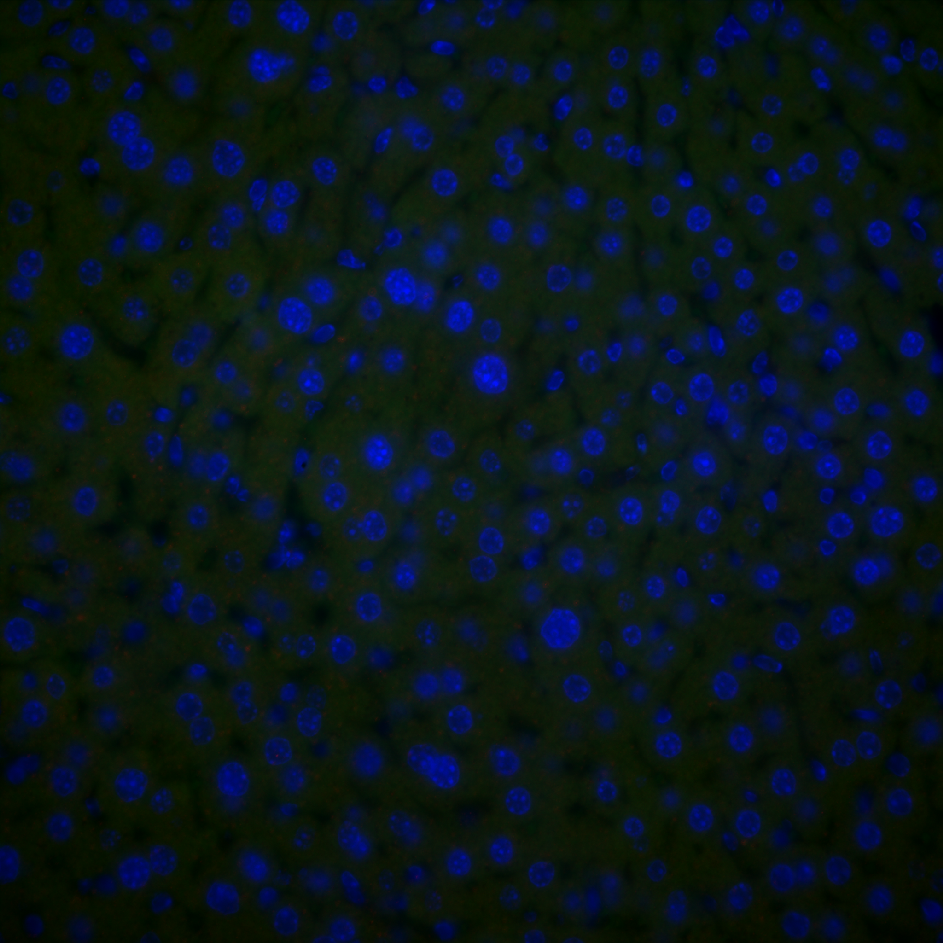

Supplement: Supplementary file 4 — Source Data Fig. 3 [file 44321_2023_21_MOESM4_ESM.zip › Figure 03 SourceData/Fig03PanelC/Mouse#151-40x-GRB.tif]

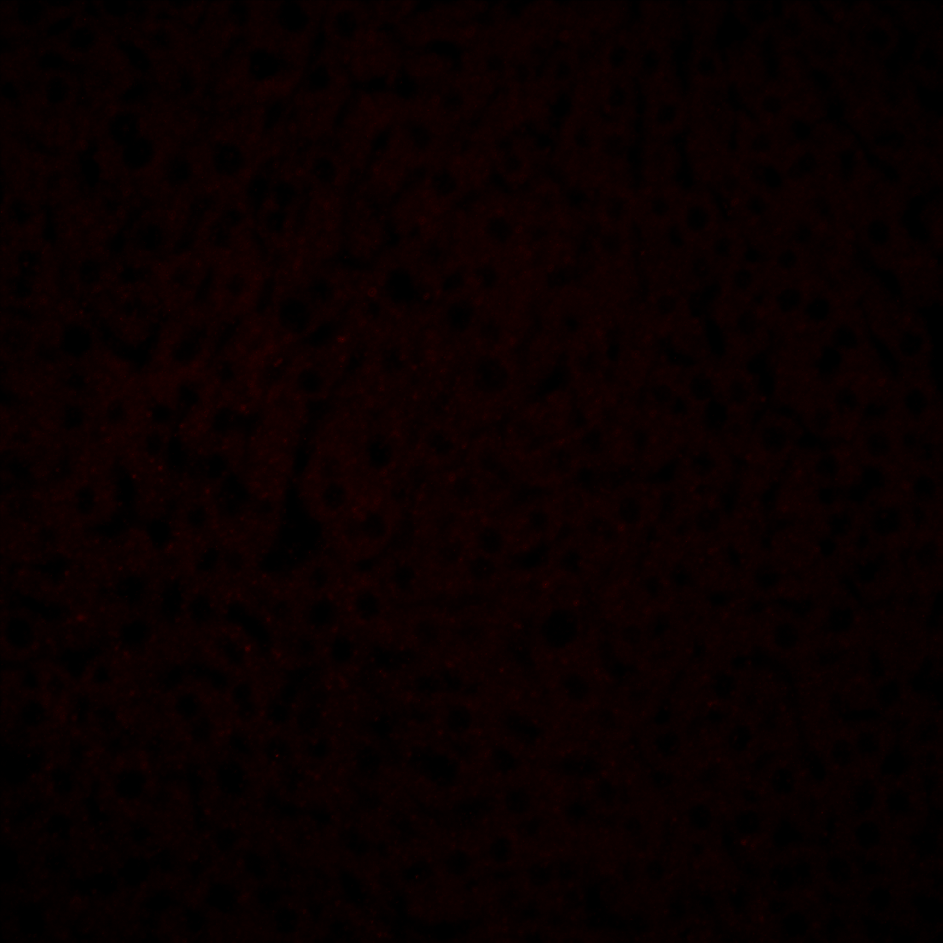

Supplement: Supplementary file 4 — Source Data Fig. 3 [file 44321_2023_21_MOESM4_ESM.zip › Figure 03 SourceData/Fig03PanelC/Mouse#151-40x-R.tif]

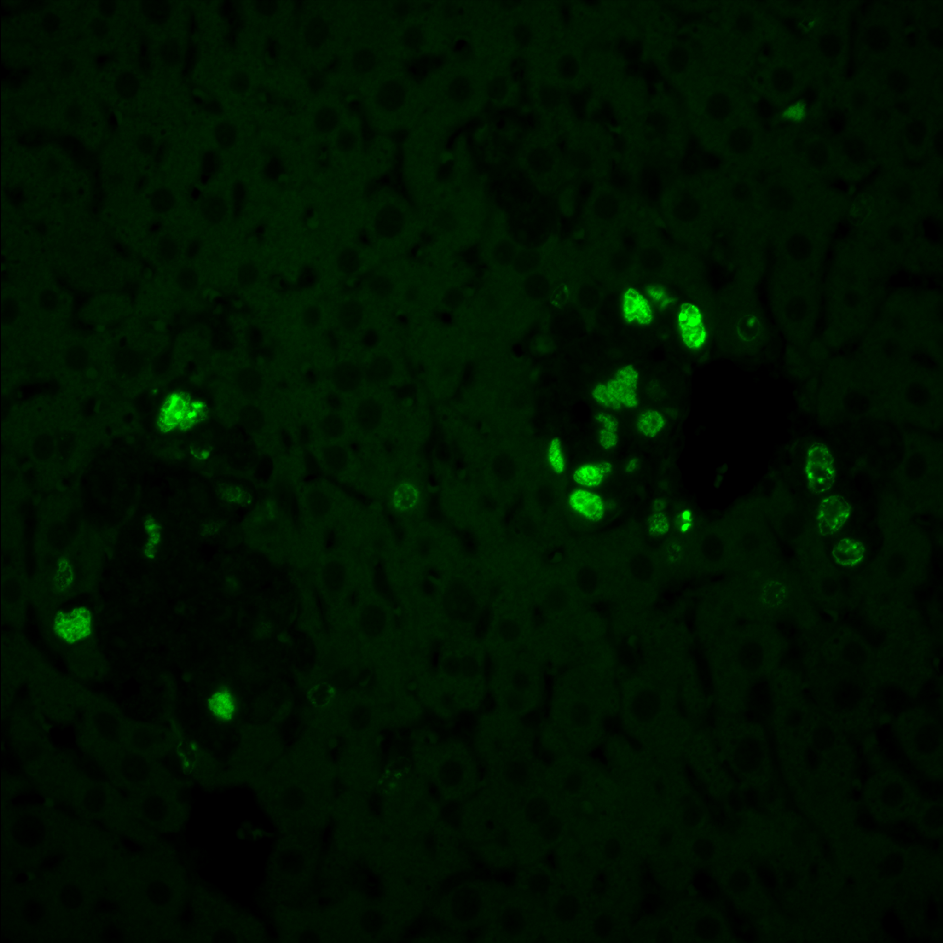

Supplement: Supplementary file 4 — Source Data Fig. 3 [file 44321_2023_21_MOESM4_ESM.zip › Figure 03 SourceData/Fig03PanelC/Mouse#156-40x-G.tif]

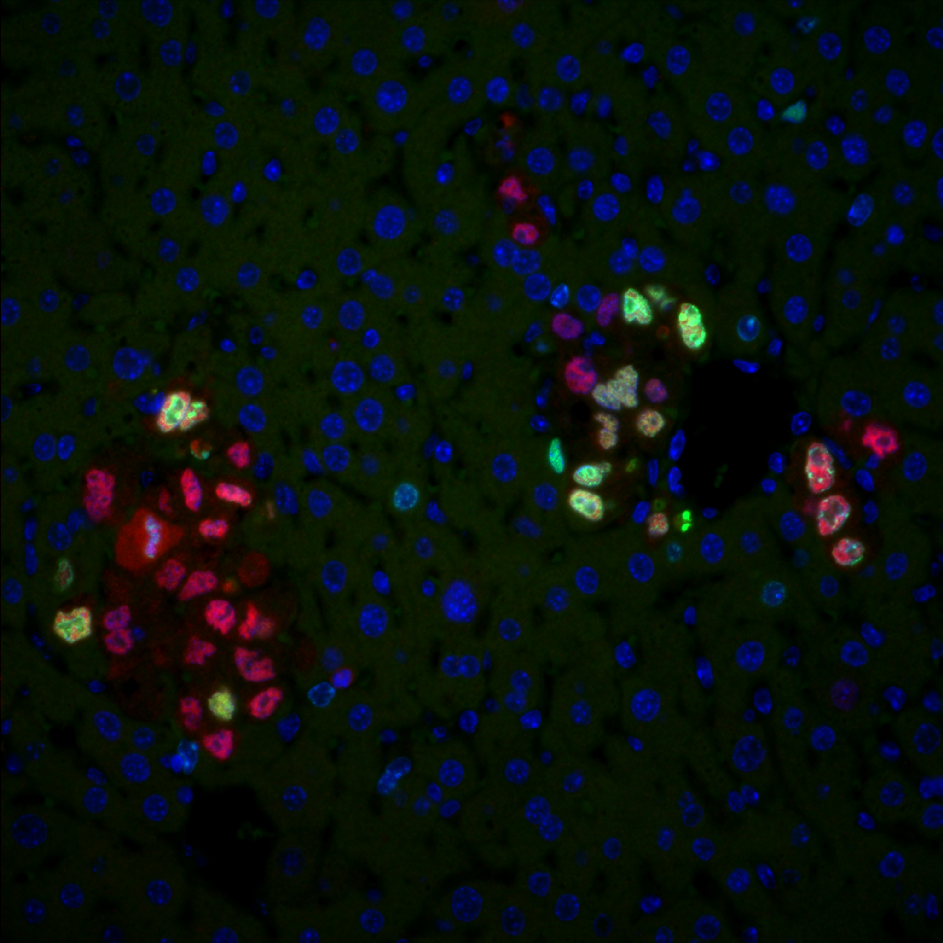

Supplement: Supplementary file 4 — Source Data Fig. 3 [file 44321_2023_21_MOESM4_ESM.zip › Figure 03 SourceData/Fig03PanelC/Mouse#156-40x-GRB.tif]

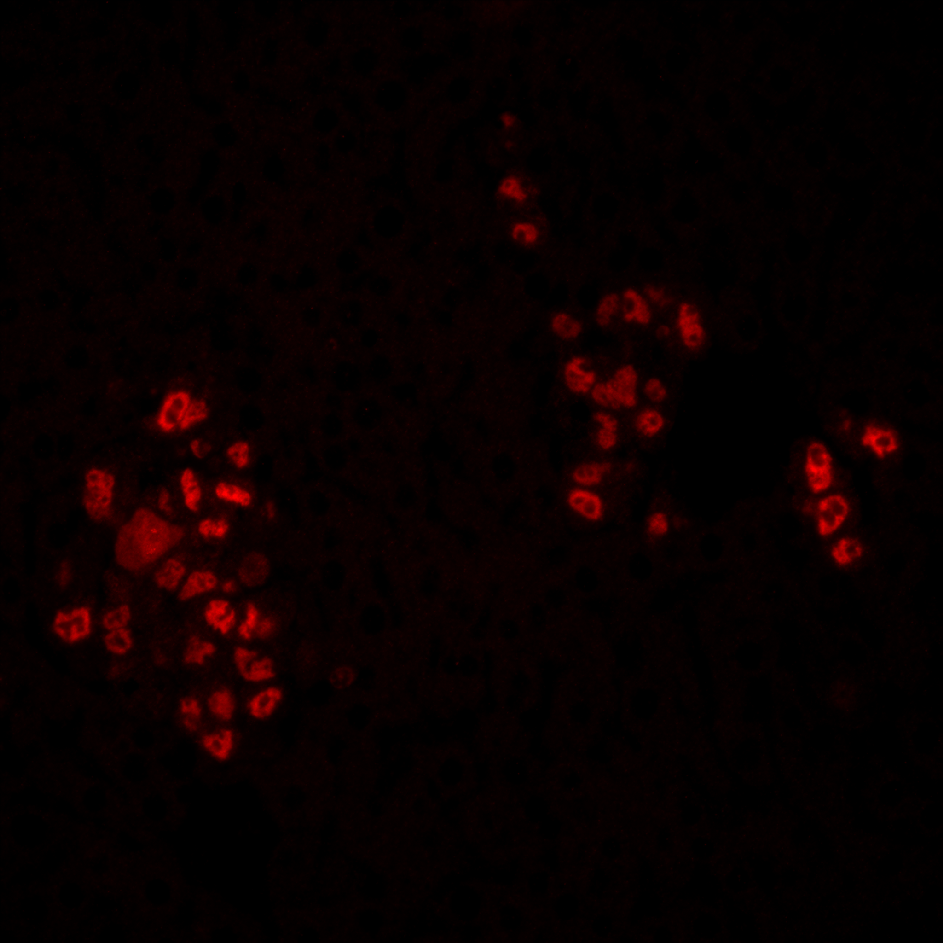

Supplement: Supplementary file 4 — Source Data Fig. 3 [file 44321_2023_21_MOESM4_ESM.zip › Figure 03 SourceData/Fig03PanelC/Mouse#156-40x-R.tif]

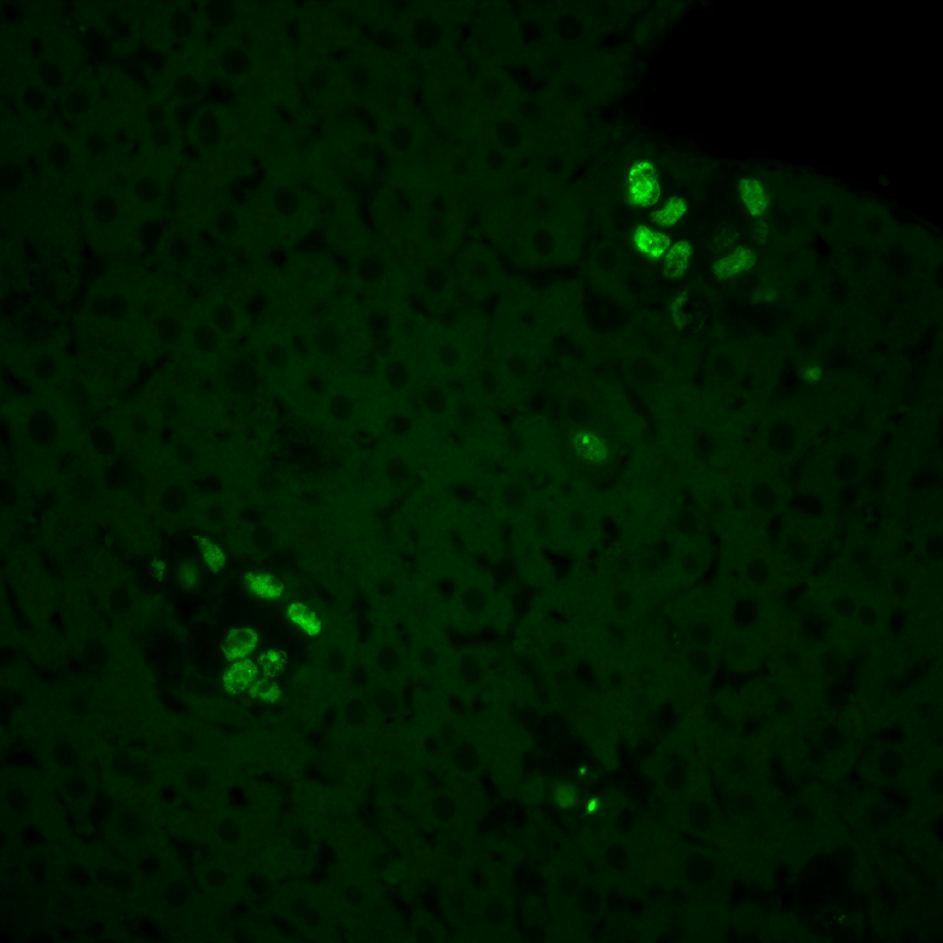

Supplement: Supplementary file 4 — Source Data Fig. 3 [file 44321_2023_21_MOESM4_ESM.zip › Figure 03 SourceData/Fig03PanelC/Mouse#159-40x-G.tif]

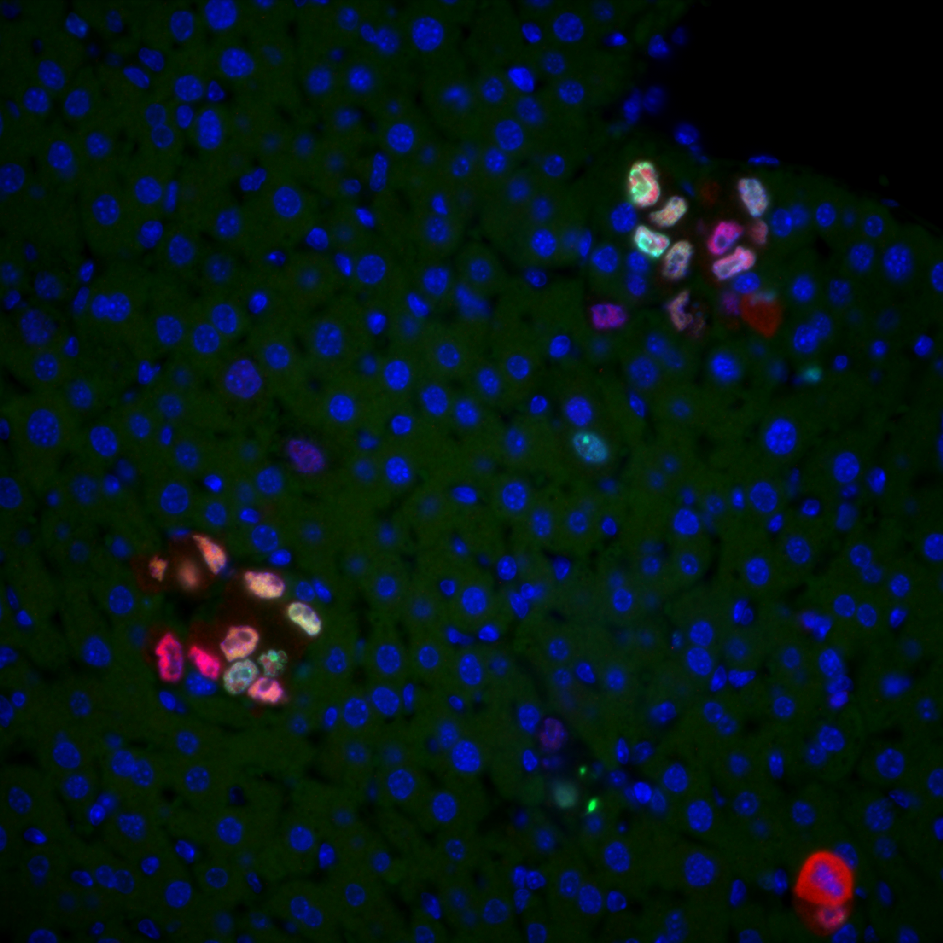

Supplement: Supplementary file 4 — Source Data Fig. 3 [file 44321_2023_21_MOESM4_ESM.zip › Figure 03 SourceData/Fig03PanelC/Mouse#159-40x-GRB.tif]

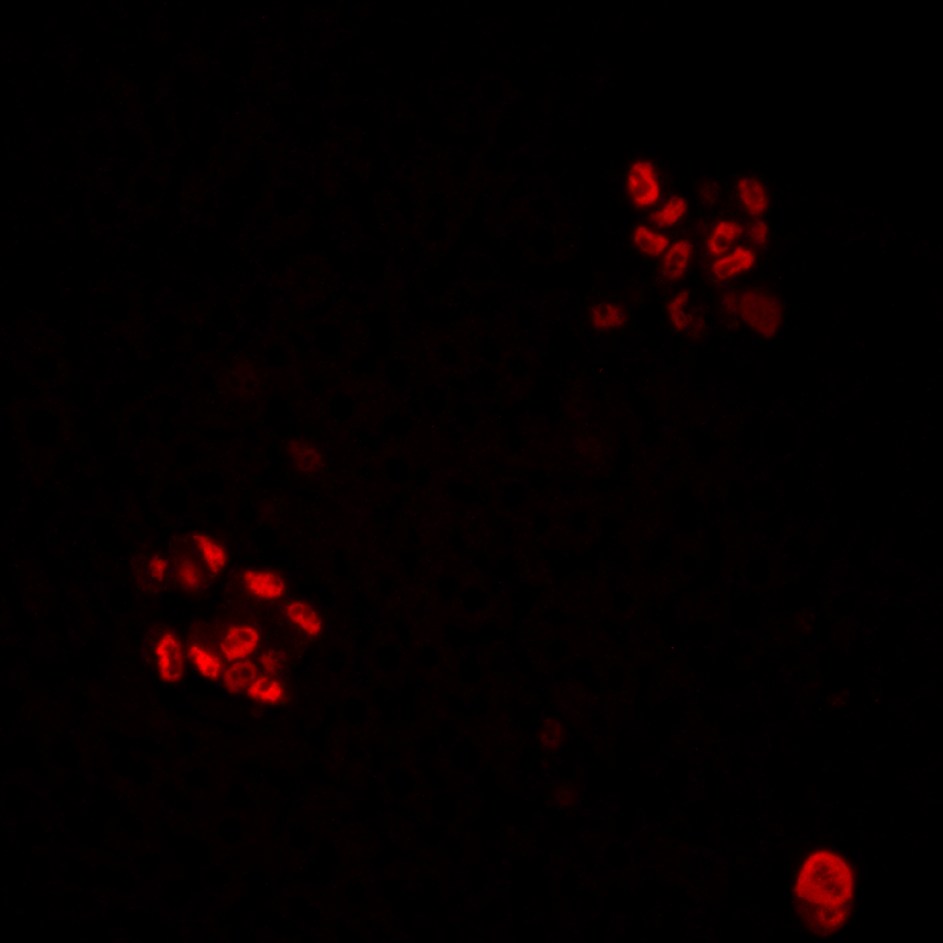

Supplement: Supplementary file 4 — Source Data Fig. 3 [file 44321_2023_21_MOESM4_ESM.zip › Figure 03 SourceData/Fig03PanelC/Mouse#159-40x-R.tif]
