# Supplementary material for: FGF19 and its analog Aldafermin cooperate with MYC to induce aggressive hepatocarcinogenesis
Source: EMBO Mol Med. 2024 Jan 16;16(2):2. doi: 10.1038/s44321-023-00021-x (PMC10897482; doi:10.1038/s44321-023-00021-x)
Supplement: Supplementary file 4 — Source Data Fig. 3 [file 44321_2023_21_MOESM4_ESM.zip › Figure 03 SourceData/Fig03PanelA/PurificationAldafermin Step#2.pdf]

BioLogic DuoFlow Run Report

Project: Purif FGF19 (Guillaume)
Method: HisTrap Nickel 1mL (30mL)
Run: PurifProt#9 Step#2

User: Olivier **Run Date:** 04:54:25 PM 05-30-23

Printed: *jeudi 30 novembre 2023*

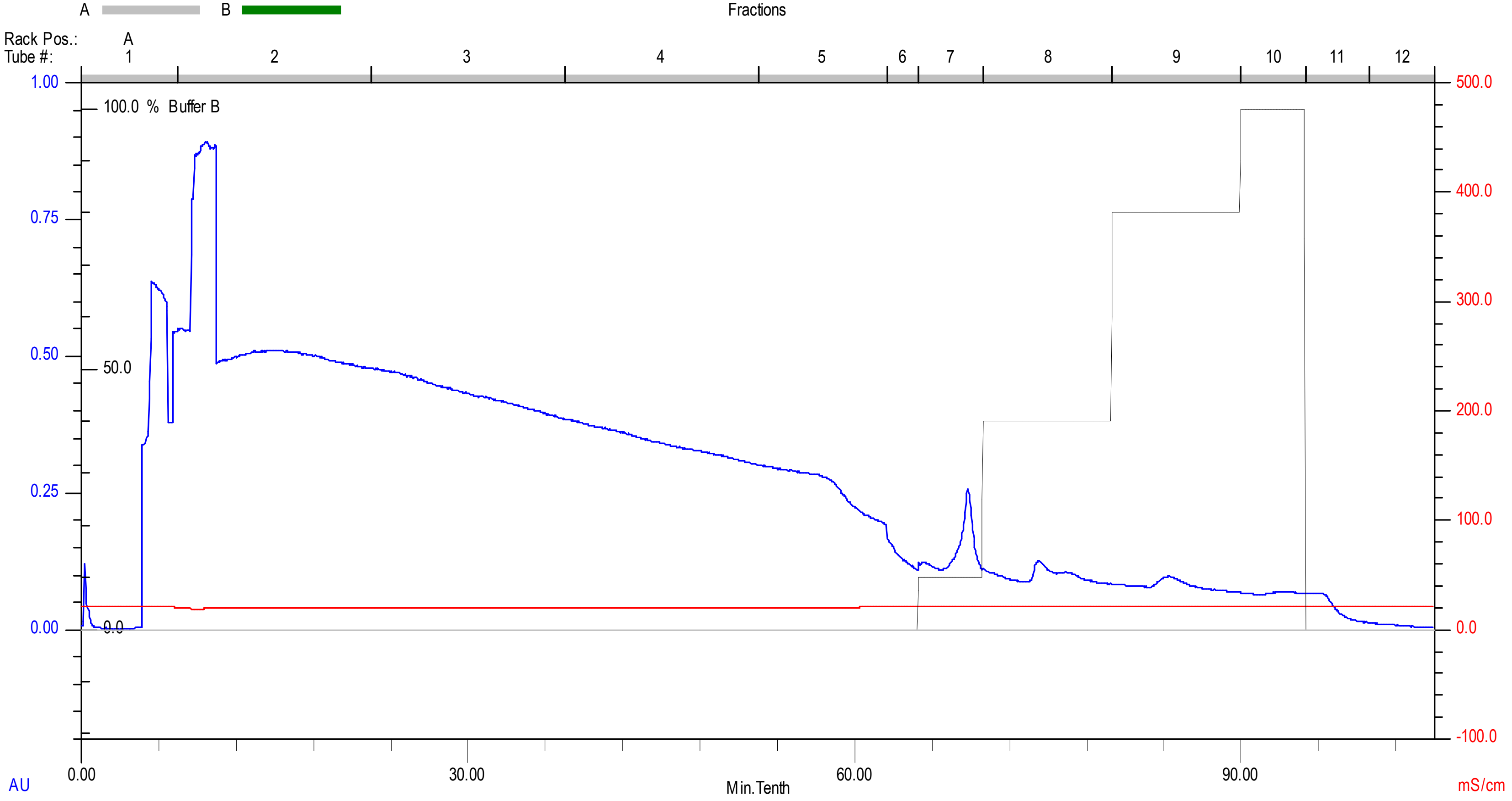

Line style	Detector	Base	Max	Units
	UV	0.00	1.00	AU
	Conductivity	-0.0	500.0	mS/cm
	GP pressure	0.0	4000.0	psi
	% Buffer B	0.0	105.0	% Buffer B

BioLogic DuoFlow Run Report

User: Olivier

Run Date: 04:54:25 PM 05-30-23

Printed: *jeudi 30 novembre 2023*

Project: Purif FGF19 (Guillaume)

Method: HisTrap Nickel 1mL (30mL)

Run: PurifProt#9 Step#2

Sample:

Column:

Buffer A: Buffer A

Buffer B: Buffer B

Operator: Olivier

Flow Rate:

Gradient:

Fraction Size:

Method Description:

Run Description:
