# Supplementary material for: FGF19 and its analog Aldafermin cooperate with MYC to induce aggressive hepatocarcinogenesis
Source: EMBO Mol Med. 2024 Jan 16;16(2):2. doi: 10.1038/s44321-023-00021-x (PMC10897482; doi:10.1038/s44321-023-00021-x)
Supplement: Supplementary file 6 — Expanded View Figures [file 44321_2023_21_MOESM6_ESM.pdf]

Expanded View Figures

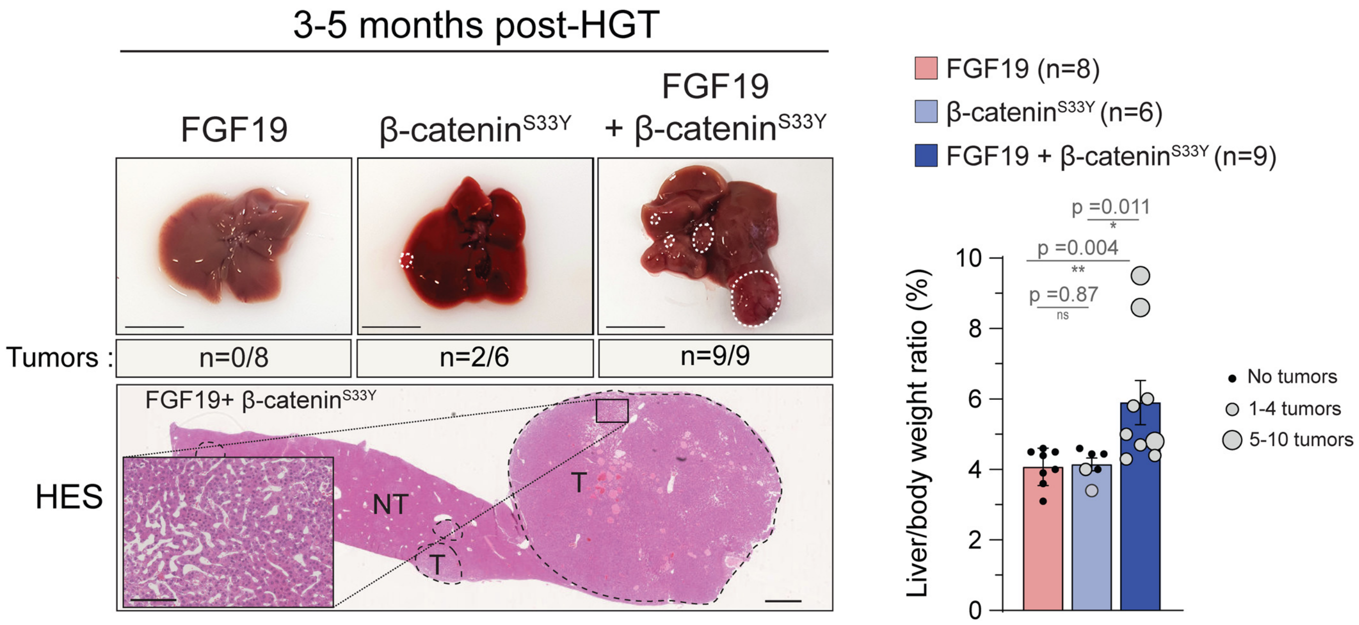

Figure EV1. FGF19 cooperates with β-catenin^{S33Y} to induce hepatic carcinogenesis.

Representative livers and HES-stained sections from mice following hydrodynamic gene transfer with either FGF19, β-catenin^{S33Y} or both. Tumor incidence is indicated. Liver/body weight ratio for each mouse, with dot sizes proportional to tumor burden. Scale bar for zoom: 200 µm. Scale bar for large view: 1 cm. Mann–Whitney test statistical significance is indicated. Data are represented as mean ± SD. ns (non significant) $p > 0.05$, *$p < 0.05$, **$p < 0.01$.

 © The Author(s)

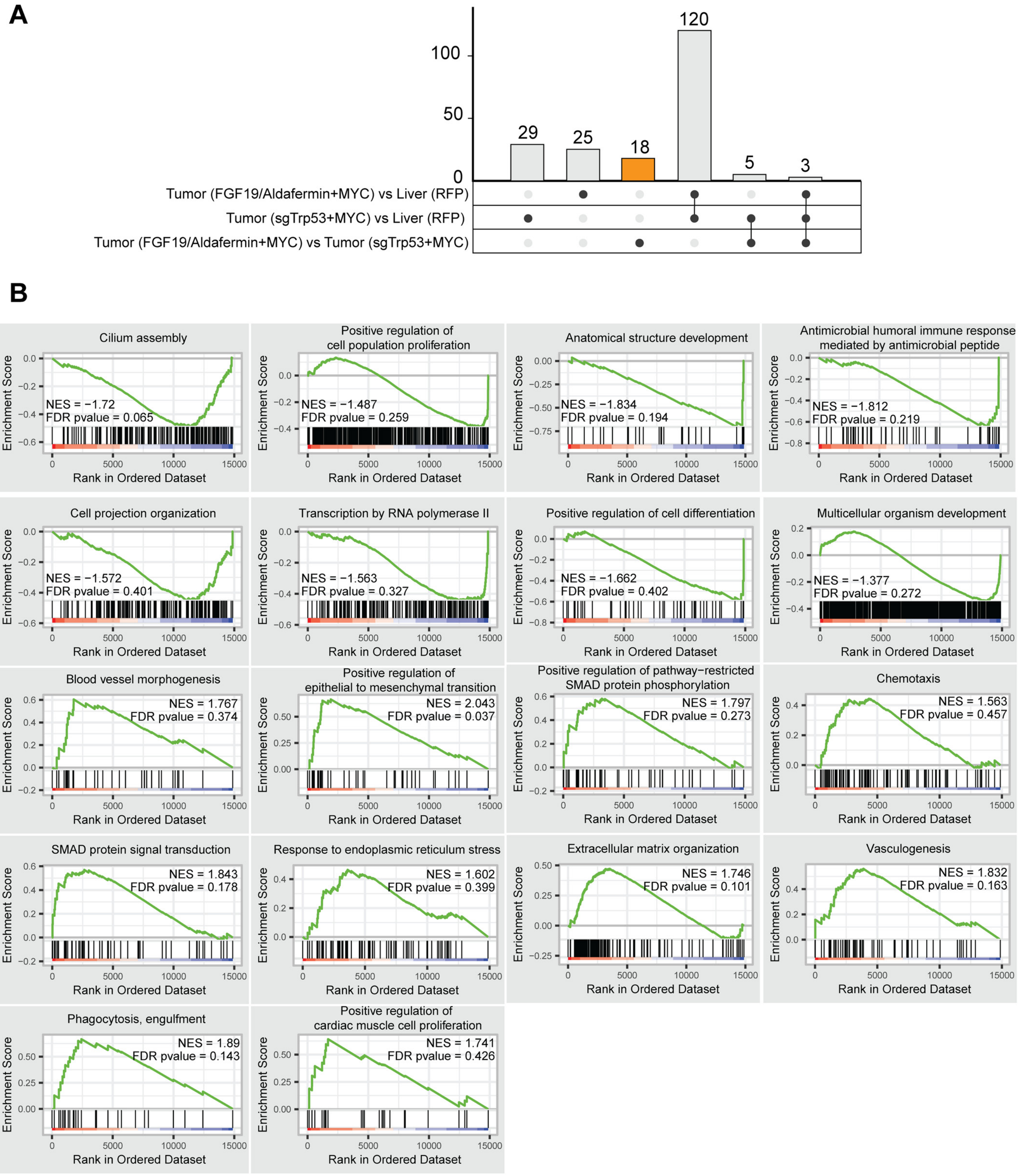

Figure EV2. GSEA Pathways enriched in FGF19/Aldafermin + MYC driven tumors.

(**A**) Upset plot depicting the number of unique and shared GSEA pathways enriched in each sample groups analyzed by RNAseq. FGF19 + MYC and Aldafermin + MYC samples were pooled for this analysis. (**B**) GSEA analysis of the 18 pathways specifically enriched in FGF19/Aldafermin + MYC vs sgTrp53 + MYC tumors. Kolmogorov–Smirnov statistical test significance is indicated. Data information: Threshold of significance for GSEA: $p < 0.01$. Complementary data are provided in Dataset EV1.

© The Author(s)
